# Pluripotent stem cells for disease modeling and drug screening: new perspectives for treatment of cystic fibrosis?

**DOI:** 10.1186/s40348-015-0023-5

**Published:** 2015-12-14

**Authors:** Ulrich Martin

**Affiliations:** Leibniz Research Laboratories for Biotechnology and Artificial Organs (LEBAO), Department of Cardiothoracic, Transplantation and Vascular Surgery, Hannover Medical School, Hannover, Germany; REBIRTH Cluster of Excellence, Hannover, Germany; Biomedical Research in Endstage and Obstructive Lung Disease Hannover (BREATH), Member of the German Center for Lung Research (DZL), Hannover Medical School, Hannover, Germany

**Keywords:** Cystic fibrosis, Induced pluripotent stem cells, Drug screening, Cell therapy

## Abstract

Despite continuous improvements in treating clinical symptoms and the identification of single compounds that effectively rescue some rare mutations in the cystic fibrosis transmembrane conductance regulator (CFTR), associated lung and liver pathologies remain largely untreatable and no real breakthrough is visible for the majority of patients suffering from cystic fibrosis (CF).

Novel compounds have to be identified and tailored in combination to specific CFTR mutations, to different tissues, or even to the individual patient. Immortalized cell lines overexpressing mutant CFTR are typically used to screen candidate molecules but have proven to be poor predictors of clinical efficacy. The complexity of CFTR maturation and turnover requires the use of cellular models that closely recapitulate the specific properties of the clinically most affected organs. Importantly, current screening efforts based on primary airway cells or intestinal organoids cannot specifically target single rare CFTR mutations or mimic multiple cell types.

In the near future, genetically engineered induced pluripotent stem cells will provide an excellent basis for personalized organotypic models of CF disease and biological screens for identification of CFTR potentiators and correctors.

## Background

Regardless of substantial clinical progress, cystic fibrosis (CF) is still a fatal congenital disease, affecting more than 70,000 patients worldwide, with high morbidity and a limited lifespan. Health care costs are estimated to be 3–4 billion € per year. Despite decades of intense research, gene therapy of CF did not enter clinical routine. Low transduction efficiencies in airway cells, immune reactions, and failure to achieve long-term expression in vivo prevented broader clinical application. Importantly, although the recent identification of cystic fibrosis transmembrane conductance regulator (CFTR) correctors and potentiators is promising, no compounds are available thus far which show significant clinical benefit in all F508del-carrying patients. In the case of other, more rare but substantial trafficking defect mutations (http://www.cftr2.org), which also cause severe CF lung pancreatic and liver involvement, existing correctors have not yet been shown to be effective. Thus, novel therapeutic concepts, as well as correctors showing clinical benefit and leading to a normal lifespan and the capacity to participate in social and economic life for patients carrying such mutations would entail substantial benefit for the community as a whole.

## Is there a need for stem cell-based drug screening?

Nearly 2000 CFTR gene mutations are known, of which ~130 are pathogenic, impairing its translation, cellular processing, and/or chloride channel gating. Therefore, small molecule therapy restoring function to mutant CFTR is a priority in the field. High throughput (HT) screens have identified CFTR potentiators, which restore the channel activity by enhancing gating [1, 2], and correctors, which rescue the most frequent trafficking mutant (F508del) to the cell surface in vitro. The potentiator Ivacaftor (VX-770) was approved by US Food and Drug Administration/European Medicines Agency (FDA/EMA) in 2013 for G551D and recently for further eight gating mutations. However, these mutations are present in just 4–5 % of all CF patients. For most CF patients, an effective small molecule treatment is not yet available. Thus far, results from clinical trials on patients homozygous for F508del with the best known corrector drugs (VX-809/661) are modest [3]. A combination of the correctors VX-809/661 and the potentiator VX-770 did improve lung function but only to a limited extent (~4 %) [4, 5] and reportedly only in a subgroup of patients [6]. Moreover, only ~40 % of patients are F508del homozygous and the efficacy of correctors for patients with only one F508del allele is expected to be even lower. At least 15 % of all CF patients are unlikely to benefit from F508del-CFTR corrector therapies, as they lack F508del in both alleles.

It is therefore clear that in the case of F508del and other trafficking mutations, novel compounds have to be identified. Indeed, current data indicate that a combination of CFTR correctors, potentiators, and molecules that prevent an excessive turnover of mutant proteins will be required [7]. This combination ideally should be tailored not only to specific CFTR mutations but also to the individual patient, who in most cases presents two different mutations. Furthermore, the complexity of the mutant CFTR maturation and turnover kinetics requires the use of advanced cellular models that closely recapitulate the properties of the most affected organs (lung, bile duct, pancreas, and intestine). Ideally, this should be implemented at the screening stage to rapidly filter out compounds that are ineffective or toxic to the human native epithelium. However, primary culture techniques are cumbersome and, despite recent progress [8], airway cells of rare mutants, which are usually available only through bronchial brushes, will probably not provide enough material for HT assays. Also, it has to be emphasized that current large screening initiatives based on primary airway cells are not able to target rare CF mutations, since explanted lung tissue homozygous for rare mutations is generally not available. Furthermore, the genetic engineering of differentiated primary cells to establish appropriate reporter lines is extremely difficult.

Most screens have therefore been performed using immortalized cell lines overexpressing CFTR mutants and halide indicators [9]. Only the most promising compounds were validated on primary human bronchial epithelial cells and showed highly variable and limited correction. However, the validation of compounds in stable organotypic cell systems at an early stage is essential, as immortalized cell lines do not show the physiological characteristics of the relevant respiratory, intestinal, pancreatic, or bile duct epithelia, including regulation of CFTR expression, traffic, and function, in particular in the context of inflammation and tissue injury responses.

It is therefore not surprising that immortalized cell lines overexpressing mutant CFTR variants are poor predictors of clinical efficacy and that alternative screening methods are required. It is consensus that those HT and medium throughput (MT) assays which have been used for identification of existing potentiators and modulators are suboptimal, in particular because all of them relied on immortalized cell lines overexpressing mutant CFTR variants without considering the patient’s genomic background and specific cellular and molecular characteristics of primary airway and bile duct epithelium.

Addressing at least some of these limitations, a method to propagate intestinal organoids from individual CF patients was recently developed, which is now used as a novel, individualized screening platform for small molecules [10]. Certainly this represents a major step forward, and intestinal organoids grown from gut biopsies apparently closely reflect the properties of CF disease intestinal epithelium. On the other hand, it has not yet been firmly shown that intestinal organoids directly recapitulate the functional characteristics of diseased airway epithelium and that CFTR activity in these organoids is an accurate predictor of CF lung disease, the most serious cause of morbidity and mortality in CF. Notably, also progressive pancreas and liver pathology caused by the plugging of pancreatic and biliary ducts present serious and frequent complications, which are untreatable using currently available interventions [11]. Clearly, it is not obvious that compounds validated in the intestinal model, which presents only partially differentiated enterocytes, will cover all aspects of CFTR trafficking correction.

## Induced pluripotent stem cells for drug screening and disease modeling

Until recently, screening activities aiming at the identification of novel correctors and potentiators were hampered by the lack of a suitable source of expandable patient-derived cells that can be easily genetically engineered. These limitations were recently overcome by two groundbreaking developments: In 2006, Yamanaka demonstrated the possibility to reprogram somatic cells into the so-called induced pluripotent stem cells (iPSCs) [12], a finding that was awarded the Nobel Prize in Medicine in 2012. And probably as important, novel technologies for efficient targeted genome engineering using designer nucleases such as Transcription Activator-like Effector Nucleases (TALENs) or the Clustered Regularly Interspaced Short Palindromic Repeats (CRISPR) system opened the opportunity for specific correction and introduction of mutations as well as efficient targeted insertion of transgenes [13].

Meanwhile, the generation of human iPSCs has become a standard procedure in many laboratories and it is now clear that these cells are almost indistinguishable from embryonic stem cells (ESCs) with respect to their phenotype, culture characteristics, and potential for proliferation and differentiation [14]. Remarkably, low reprogramming efficiencies are not critical anymore with reported efficiencies of up to 100 % [15]. In addition, reprogramming of a variety of cell types was demonstrated. Whereas invasive skin biopsies for isolation of fibroblasts were initially required, nowadays iPSCs can be isolated from hair bulks or blood as easily accessible cell sources [16–18].

From a practical standpoint and industrial perspective, it was mandatory to address further issues that are critical for large-scale application of iPSC derivatives in drug screening and safety pharmacology. This includes the hitherto inability to produce large cell masses of iPSC in defined culture media. As for reprogramming efficiencies, major progress could be achieved also in this field and our group was able to demonstrate the possibility of long-term expansion of human-induced pluripotent stem cells in scalable suspension culture under defined conditions [19, 20]. Meanwhile, large numbers of human iPSCs can be produced in fully controlled stirred bioreactors [21].

The lack of robust and efficient differentiation protocols enabling the directed derivation of specific cell lineages has been another limiting factor for the development of stem cell-based therapies. However, sequential inhibition and activation of molecular differentiation pathways now allow a targeted and efficient differentiation of human pluripotent stem cells into various lineages. Of special relevance for CF, hiPSC-derived bile duct epithelium can be obtained if suitable differentiation protocols are applied [22]. Also, remarkable progress was achieved during recent years concerning the differentiation of pulmonary cells. As a prerequisite for generation of mature airway cells, efficient derivation of definitive endoderm and anterior foregut endoderm from murine and human pluripotent stem cells was achieved through initial activation of nodal signaling by Activin A and subsequent dual inhibition of transforming growth factor (TGF)-β and bone morphogenic protein (BMP) signaling applying the chemical compounds dorsomorphin, IWP2, and SB431542 [23–25]. Subsequent treatment with Wnt3a or CHIR, a chemical Wnt-agonist, fibroblast growth factor 10 and bone morphogenetic protein 4 led to ventralization and targeted generation of early lung progenitors [24, 26, 27]. Also, key factors for further specification of different respiratory lineages including keratinocyte growth factor, dexamethasone, and cAMP-elevating agents were identified [24, 26–29]. It is noteworthy that small organic molecules as a cheap alternative with fewer lot-to-lot variations are increasingly replacing recombinant proteins in such protocols [30]. In case of cardiomyocytes, our group already succeeded in the development of highly efficient, scalable protocols that are exclusively based on chemical compounds and thus are relatively inexpensive and robust and result in dramatically improved cardiac differentiation efficiencies of up to 95 % in fully controllable stirred bioreactor systems [31], enabling the production of the vast numbers of cardiomyocytes required for drug screening and clinical cell therapy. However, respiratory differentiation protocols are more complex and as yet replacement of recombinant proteins could be achieved only partially.

In contrast to primary bronchial cells, which presently provide the standard in differentiated airway cell culture, iPSCs show an unlimited potential for proliferation and differentiation. Also of relevance in particular for automated high throughput drug screening is the possibility to apply novel site-specific gene editing in hiPSCs including TALENs [13]. This opens up the unique possibility to generate patient and CFTR mutation-specific cell lineages, carrying halide- or voltage-sensitive fluorescent proteins for functional screens or reporter tags within the endogenous CFTR gene for direct visualization of trafficking. Clearly, such opportunities will constitute an important step to run high throughput screening of chemical libraries.

Remarkably, while gene editing in intestinal organoids that are generated based on patient-specific intestinal stem cells from gut biopsies currently requires antibiotic selection to obtain transgenic stem cell clones, footprintless gene editing of isogenic iPSC lines has been proven feasible through TALENs/single-stranded oligonucleotides (ssODNs) without antibiotic selection or FACSorting [13]. This offers not only the opportunity to generate isogenic control lines by correcting or introducing mutations into the CFTR gene but also to edit potential genomic modifiers of CF disease such as cytokeratin 8, Syntaxin 1A, or ets homologous factor [32–34]. Importantly, this also provides the opportunity to investigate the isolated effects of rare mutations in cases where no homozygous donors are available. The second CFTR allele of iPSCs from heterozygous donors or compound heterozygotes can be fully inactivated through introduction of sequence-specific deletions or transgene integrations utilizing designer nucleases such as TALENs or CRISPR/Cas. In the resulting engineered cells, isolated functional measurement of the remaining allele carrying the rare mutation of interest is possible.

**Fig. 1:**
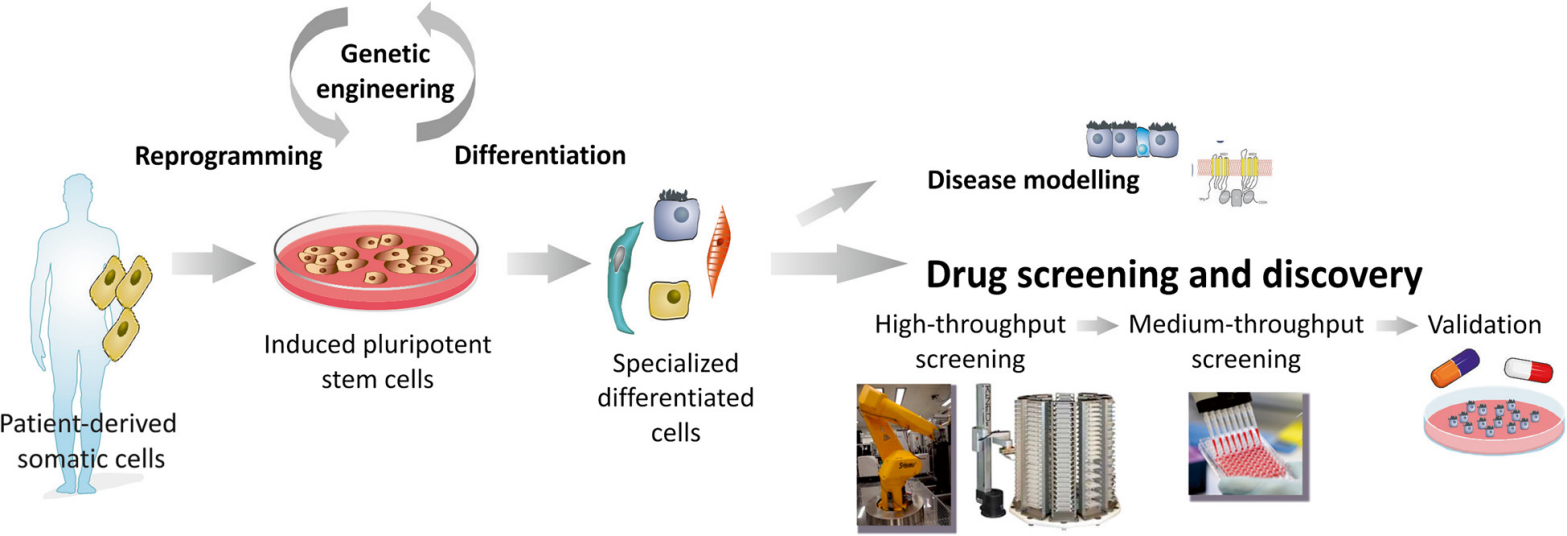
Generation and use of CF-disease-specific iPS cells for drug screening and disease modeling. Adult somatic cells (e.g., from blood) from any patient can be reprogrammed into induced pluripotent stem (iPS) cells. These cells can be genetically modified, expanded, and differentiated into the cell types of interest and used (**i**) to understand the molecular mechanisms underlying disease phenotypes, for example, the molecular causes for different clinical phenotypes in CF patients with similar mutations, and (**ii**) in drug screening and discovery, to determine the effects of candidate drugs and new compounds, and to identify target pathways

Genetically engineered reporter iPSCs can finally be differentiated into CF relevant cell types such as respiratory [28], pancreatic [35], and bile duct epithelium [22] and utilized for HT screens of small molecules. Also, these iPSC derivatives can be used for the further validation of compound candidates or combinations of compounds in novel electrophysiological and cell culture assays that probe more distant parameters, such as mucus secretion, bioactive lipid metabolism, inflammation, and tissue remodeling. Importantly, and in contrast to current screening initiatives based on primary cells or intestinal organoids, the use of CF disease-specific iPSCs generally offers the unique possibility to validate promising compounds not only in the cell type used in the primary screen but also in addition in other organotypic epithelia (Fig. 1).

## Potential hurdles and technical limitations for application of iPS cells in disease modeling and drug screening

The impressive progress in the generation and differentiation of iPSCs and in targeted genome engineering clearly provides manifold new opportunities for disease modeling and drug screening. In fact, there are already various examples for the decipherment of pathomechanisms especially of cardiovascular [36] and neurological disorders using patient-specific iPSCs [37]. Also, the pharmaceutical industry has recognized the potential and the advantages of iPSCs for drug screening and safety pharmacology and has already made substantial investments in collaborative efforts with academia and the development of own R&D units in this evolving field (see, for example, EU’s Innovative Medicines Initiative).

On the other hand, it has to be emphasized that there are still various technology-related imponderables and critical hurdles to overcome. One of these hurdles is the lack of robustness of most differentiation protocols. Even in case of the most efficient targeted differentiation approaches that rely on fully defined media and application of small organic compounds, the success and efficiency is not always reproducible. Even though it is obvious that failure of individual preparations is frequently based on the quality of the starting stem cell population, it is still barely possible to exclude such failure. Clearly, resulting variations in the composition and purity of the target cell population significantly complicate the conduction of high throughput screens and their routine application in drug validation and safety pharmacology.

As discussed above, one major advantage of disease modeling and biological screens based on iPSCs is the availability of patient-specific cells with defined genetic background that theoretically allows the direct correlation of the observed cellular phenotype with clinical data. However, it is increasingly recognized that individual iPSC clones generated from one donor can display a high degree of variation in culture and differentiation characteristics that can sometimes even exceed phenotypic differences between iPSC clones of different donors.

Moreover, the experimental requirement for suitable control lines poses another problem: Because of the well-known influence of genetic modifiers and the individual genetic background even in so-called monogenic diseases, it is meanwhile considered state-of-the-art that isogenic gene-corrected iPSC lines are used. It is noteworthy, however, that although the availability of modern genome engineering approaches renders this technically feasible, it is increasingly recognized that the single cell cloning procedures required to select genetically engineered cells may result in iPSC clones with properties different from the original cells.

In general, and although underlying mechanisms are widely unknown, altered characteristics of individual iPSC clones are likely due to culture adaptation and selection of (epi)genetic subclones not only during genetic engineering but also during iPSC generation, single cell cloning, and iPSC expansion. At this point, it is still unclear whether such differences affect only culture and differentiation behavior or may extend to functional properties of differentiated derivatives. Therefore, it remains to be investigated, whether the use of gene-corrected isogenic control lines is sufficient to overcome clonal effects on cellular assays designed to mirror a disease of interest in the dish.

## Conclusions

The use of genetically engineered patient-specific iPS cells can be considered as a highly innovative and valuable new platform not only for a better understanding of the different CF disease phenotypes but also for the identification of drugs that are able to functionally correct the organ-specific consequences of the different classes of common and rare CFTR mutations of wide clinical applicability.
